# Stable Isotopes of C and N Reveal Habitat Dependent Dietary Overlap between Native and Introduced Turtles *Pseudemys rubriventris* and *Trachemys scripta*

**DOI:** 10.1371/journal.pone.0062891

**Published:** 2013-05-13

**Authors:** Steven H. Pearson, Harold W. Avery, Susan S. Kilham, David J. Velinsky, James R. Spotila

**Affiliations:** 1 Drexel University, Department of Biodiversity, Earth and Environmental Science, Philadelphia, Pennsylvania, United States of America; 2 Drexel University, Department of Biology, Philadelphia, Pennsylvania, United States of America; University of Kent, United Kingdom

## Abstract

Habitat degradation and species introductions are two of the leading causes of species declines on a global scale. Invasive species negatively impact native species through predation and competition for limited resources. The impacts of invasive species may be increased in habitats where habitat degradation is higher due to reductions of prey abundance and distribution. Using stable isotope analyses and extensive measurements of resource availability we determined how resource availability impacts the long term carbon and nitrogen assimilation of the invasive red-eared slider turtle (*Trachemys scripta elegans*) and a native, threatened species, the red-bellied turtle (*Pseudemys rubriventris*) at two different freshwater wetland complexes in Pennsylvania, USA. At a larger wetland complex with greater vegetative species richness and diversity, our stable isotope analyses showed dietary niche partitioning between species, whereas analyses from a smaller wetland complex with lower vegetative species richness and diversity showed significant dietary niche overlap. Determining the potential for competition between these two turtle species is important to understanding the ecological impacts of red-eared slider turtles in wetland habitats. In smaller wetlands with increased potential for competition between native turtles and invasive red-eared slider turtles we expect that when shared resources become limited, red-eared slider turtles will negatively impact native turtle species leading to long term population declines. Protection of intact wetland complexes and the reduction of introduced species populations are paramount to preserving populations of native species.

## Introduction

Habitat degradation is the leading cause of extinction and population declines worldwide [1]. Species richness and species diversity generally decrease as habitat availability is reduced and rates of disturbance increase [2], [3]. For species in the same guild of an ecological community, decreases in resource availability can lead to increases in resource overlap and a narrowing of niche breadth [4], [5] leading to increased risk of resource competition [6]. Competition for shared resources between species often negatively impacts the growth rates, fecundity rates and/or survivorship of at least one of the competing species [7]. Disturbed habitats are susceptible to the establishment of introduced species due to alteration of community structure with open niches that can be filled by non-native species [8], [9].

Today, naturally evolved and established ecological communities are being disrupted at unprecedented rates through habitat degradation and species introductions [1], leading to alterations in resource availability and changes in community structure [2]. Native species are negatively impacted by introduced species through predation and competition [10]. Introduced predators can cause the severe collapse of native faunas that do not adapt quickly enough to increased predation rates [11], [12]. Introduced competitors cause decline of native species by increasing rates of exploitative and interference competition [7], [13]. When competition occurs for limited resources the species that more efficiently utilizes resources will competitively exclude the less efficient species [14], [15]. Co-existence between competing species can occur if inferior competitors disperse more rapidly or utilize resources that shift in space and time [16]. Competition between species may result when dietary resources are not partitioned and will cause reduced fitness levels of one or all competing species [7].

Ecological studies of diets have historically relied on short term dietary intake through observations of feeding and/or the collection of stomach contents through fecal collection, stomach flushing or dissection [17]–[19]. Long term diets of organisms have been studied through the analyses of carbon 13 and nitrogen 15 stable isotopic fractions (δ^13^C and δ^15^N) [20]–[22]. Naturally occurring isotopic fractions of nitrogen (δ^15^N) and carbon (δ^13^C) indicate an organism's trophic level and the source of carbon assimilated from its diet, respectively [23]. The premise of all stable isotope studies of animals is that isotopes of the same element are incorporated at different rates into tissue through nutrient assimilation by an organism during digestion or other physiological processes [24], [25]. Factors affecting δ^13^C and δ^15^N isotope assimilation include tissue metabolism, trophic level, temperature, C∶N ratios in items consumed, taxonomy, body size, and an organism's form of eliminating nitrogenous waste [25],[26]. Stable isotopes have been used in determining the C and N sources in organisms' diets [27], trophic position in food webs [28] and in comparative studies of species feeding ecology between study sites [29]–[31].

We used stable isotope analyses to quantify the diets and extent of resource overlap between the native red-bellied turtle (*Pseudemys rubriventris*) and the introduced red-eared slider turtle (*Trachemys scripta elegans*) in two southeastern Pennsylvania wetland complexes that differed in ecological characteristics. Red-eared slider turtles have been introduced globally and negatively impact basking behavior and growth rates of European pond turtles (*Emys orbicularis galloitalica*) and the Spanish terrapin (*Mauremys leprosa*) under experimental and natural conditions [32], [33]. We relate the results of stable isotope analyses to wetland characteristics and the potential for competition between red-bellied turtles and red-eared slider turtles.

### Study Sites

We carried out our research at two wetland complexes that differed in size, extent of connectivity, and the species richness and diversity of vegetative communities. One wetland complex was located at the Silver Lake Nature Center (SLNC), Bristol, PA and consisted of two lakes each greater than nine hectares which were connected by a creek and surrounded by protected lowland forest and parkland. The second wetland complex was at Fort Mifflin (FM), Philadelphia, PA and consisted of three small wetlands, each less than 0.8 hectares separated by steep banks and paved roads, and surrounded by mowed lawns and narrow patches of forest (Table 1).

**Table 1:** Wetland characteristics at the Silver Lake Nature Center (SLNC) and Fort Mifflin (FM), Pennsylvania, USA.

Wetland	Wetland Area	Species Richness	Shannon- Wiener Diversity Index
**SLNC**	0.21 km^2^	51	1.348
**FM**	0.04 km^2^	30	0.93

Species richness and diversity of vegetation was greater at Silver Lake Nature Center than at Fort Mifflin.

## Materials and Methods

### Ethics Statement

We collected all animals and tissue samples under the Drexel University Institutional Animal Care and Use Committee approved protocol # 18487 and Pennsylvania Fish and Boat Commission Scientific Collecting Permits # 121 issued to HWA and #345 issued to SHP. Permission to collect at SLNC and FM was granted by the land managers.

### Calculation of Wetland Size

We calculated wetland size using ArcGIS 9.3 by digitizing aquatic habitat boundaries using aerial photographs. Digitized boundaries were converted into polygons and area was calculated using Hawths Tools [34],[35]. We summed the total area of individual wetlands to calculate the total amount of aquatic habitat available for turtles to use within study sites.

### Availability of Vegetation Resources

We determined vegetative community composition through monthly vegetation surveys performed between June and September 2010. We used a hybridized quadrat-belt transect sampling technique [36]. At each wetland within a wetland complex we chose 10 littoral zone transect sites by randomly selecting 10 points along the wetland's perimeter using ArcGIS software. We located survey points using handheld Garmin GPS units and then determined final location randomly [37], [38]. Each transect was 3 m long and ran perpendicular to the wetland edge. Along each transect, three 0.5 m^2^ quadrats were sampled with 1.5 m center spacing. This sampling technique enabled determination of species composition in each quadrat and an estimate of percent cover for terrestrial plants and submerged, emergent and floating macrophytes across a 3 m gradient of water depth. We used these data to determine species richness and species diversity of riparian vegetation at each wetland studied. Species diversity was determined using the Shannon Wiener Diversity Index in which 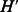 is the diversity
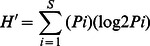
index, s = the number of species and P*i* = the proportion of total samples belonging to the i^th^ species [39].

### Sampling for Stable Isotopes

#### Sample Collection –Turtles

We captured turtles by hand, basking traps and baited hoop net traps and took tissue samples from individual sexually mature adult turtles during the active season (June through September) over a three year period (2008,2009,2010). Red-eared slider turtles are sexually dimorphic so we sampled sexually mature male red-eared slider turtles greater than 100 mm straight plastron length (SPL) and females greater than 175 mm SPL [40], [41]. Red-bellied turtles exhibit less pronounced sexual dimorphism so all turtles sampled were greater than 175 mm SPL [40], [42]. Tissues included blood drawn from the forelimb [43], tail tissue from the posterior most 3 mm of the turtle's tail and shell filings collected during ID code notching. Stable isotope sample sizes are presented in Table 2. We stored blood on ice or in a freezer for up to 12 hours until we separated blood plasma and red-blood cells by centrifugation. We took samples of tail tissue with sterile scalpel blades. Using clean half round files, we produced shell filings and collected them in sealable plastic bags. Carbon and nitrogen stable isotopes of blood tissue for red-eared slider turtles have a turnover rate of 3–6 months [44] and are representative of short term nutrient assimilation. Shell and tail tissue turnover rates are unknown for adult turtles but we assume that isotopic composition of these tissues represent diet assimilation over many years.

**Table 2:** Mean δ^13^C and δ^15^N values and sample sizes for all tissues collected from red-bellied turtle (*Pr*) and red-eared slider turtles (*Ts*) between 2008 and 2010 at the Silver Lake Nature Center (SLNC) and at Fort Mifflin (FM).

Wetland Year	Tissue Type	n		Mean δ^13^C(‰)		Mean δ^15^N (‰)		Cp-value	Np-value
		*Pr*	*Ts*	*Pr*	*Ts*	*Pr*	*Ts*		
	**Plasma**	12	5	−18.19	−25.92	6.91	9.49	**0.002**	**0.03**
**SLNC 2008**	**RBC**	10	6	−19.18	−26.63	5.56	8.33	**0.0002**	**0.004**
	**Tail**	14	7	−18.26	−24.88	6.57	9.80	**0.00007**	**0.00007**
	**Plasma**	7	6	−27.28	−26.20	11.50	12.03	0.11	0.65
**FM 2008**	**RBC**	9	5	−26.66	−25.80	10.31	9.53	0.18	0.23
	**Tail**	9	6	−26.44	−24.88	10.14	10.81	**0.018**	0.43
	**Filings**	7	6	−26.67	−25.35	11.27	10.37	**0.004**	0.26
**SLNC 2009**	**Plasma**	15	4	−19.52	−24.10	7.34	11.60	**0.029**	**0.001**
	**RBC**	12	6	−20.33	−24.30	5.97	9.98	**0.001**	**0.0009**
	**Plasma**	16	15	−26.45	−26.90	9.74	10.55	0.42	0.22
**FM 2009**	**RBC**	10	14	−27.14	−26.42	9.74	9.26	0.21	0.35
	**Tail**	10	12	−25.65	−26.91	9.63	11.12	**0.026**	**0.001**
**SLNC 2010**	**Plasma**	10	10	−18.46	−23.80	8.25	11.14	**0.0002**	**0.0003**
**FM 2010**	**Plasma**	10	9	−21.43	−24.87	10.85	11.62	0.19	0.5

P-values below the 0.05 significance level are highlighted in bold.

#### Sample Collection – Plants

We collected each plant species encountered during the monthly vegetative resource availability surveys described above. We also collected vegetation opportunistically throughout the season to ensure that we sampled all of the potential dietary items. Plants analyzed were processed as whole plants, flowers or fruits.

#### Sample Preparation and Processing

We processed turtle tissues (Table 2) following techniques described by Seminoff *et al.* [44]. All tissues were dried at 60°C for 24 to 48 hours. Vegetation samples (Table 3) were rinsed with water to ensure that animal material was removed and dried at 60°C for 24 hours. We did not extract lipids or mathematically normalize δ^13^C values because of the relatively low lipid content in the tissues we analyzed. Turtle blood has a low lipid content compared to birds for which lipid extraction of blood has been determined to be unnecessary [27], [45], [46]. Furthermore, we determined the percent lipid of tail tissue by lipid extraction with dichloromethane to be below the 5% threshold that Post et al. (2007) suggest lipid extraction or mathematical normalization of δ^13^C be performed on [47]. All samples were sealed and stored frozen until prepared for mass spectrometry. We pulverized dried samples into a homogenous powder with an agate mortar and pestle, with a glass stirring rod or with a liquid nitrogen SPEC Certiprep freezer mill. Pulverized samples weighing 0.6 mg to 1 mg for turtle tissues and 1 mg to 1.5 mg for vegetation samples were placed in 3.5×5 mm and 5×9 mm pressed tin capsules respectively, sealed and analyzed at the Patrick Center for Environmental Research, the Academy of Natural Sciences, Philadelphia, PA, using a Finnigan Delta Plus coupled to a NA2500 Elemental Analyzer (EA-IRMS). Cross contamination was avoided by cleaning all processing equipment before and after each sample. Samples were run in duplicate or triplicate and analytical variability was generally less than 3% RSD. Multiple in-house standards were analyzed for each run to assess comparability over time. Samples were reported in the standard δ (‰) notation:
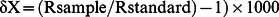
where X is either ^13^C or ^15^N and R is either ^13^C/^12^C or ^15^N/^14^N. The δ^15^N standard was air (δ^15^N = 0), and the δ^13^C standard was the Vienna PeeDee Belemnite (VPDB) limestone that was assigned a value of 0.0‰. Analytical accuracy was based on standardization of scientific grade N_2_ and CO_2_ used for continuous flow-IRMS with International Atomic Energy Agency's (IAEA) N-1, N-3, and USGS 26 for nitrogen and IAEA's sucrose, National Institute of Standards and Technology's (NIST) NBS 19, and NIST's NBS 22 for carbon, respectively.

**Table 3:** Carbon and Nitrogen stable isotope values for vegetation at Fort Mifflin (FM) and Silver Lake Nature Center (SLNC) during 2010.

Wetland Complex	Species	Common Name	Mean δ^13^C (‰)	Mean δ^15^N (‰)
	*Peltandra virginica*	Arrow Arum	−28.50	5.15
	***Lemna minor***	Duckweed	−27.85	10.09
**FM**	***Myriophyllum spp.***	Water milfoil	−16.50	2.24
	***Nuphar advena***	Spatterdock	−26.86	1.03
	*Wolffia spp*	Watermeal	−23.64	7.27
	*Amorpha fruticosa*	False Indigo	−27.13	0.20
	*Hibiscus moscheutos*	Swamp Rosemallow	−27.09	8.26
	***Lemna minor***	Duckweed	−26.49	10.08
	***Myriophyllum spp.***	Water milfoil	−24.73	9.20
	***Nuphar advena***	Spatterdock	−26.25	6.10
**SLNC**	*Parthenocissus quinquefolia*	Virginia Creeper	−28.24	5.67
	*Solanum dulcamara*	Bittersweet Nightshade	−28.24	10.53
	*Viburnum dentatum.*	Arrowwood	−26.76	5.39
	*Vitis vulpina*	Frostgrape	−26.47	7.45
	*Lyngbia spp.*	Filamentous Algae	−19.92	11.02

The values presented are the mean value for all tissue sampled from these plant species. Plants species that were sampled at both wetlands are in bold.

### Data Analysis

We analyzed results of stable isotope analysis by first averaging δ^13^C and δ^15^N values for individual samples with replicated tissues. Averaged values were then used for all subsequent analyses. We analyzed isotopic values within year and by tissue in R using standard t-tests (unequal variance assumed) with species as the grouping factor. We accepted statistical significance at the p = 0.05 level. In this study, a significant difference between species within a year was representative of isotopic niche partitioning. We analyzed isotopic values between years and by tissue using fixed effect ANOVAs with year as the treatment and isotopic means as the response variable with program R [48]. All comparisons between years were significantly different and we did not combine data between years. Significant differences between years may not be representative of dietary shifts due to the temporal variations in δ^13^C and δ^15^N signatures of aquatic vegetation [49].

## Results

### Wetland Size, Vegetative Species Richness and Vegetative Species Diversity

**Figure 1:**
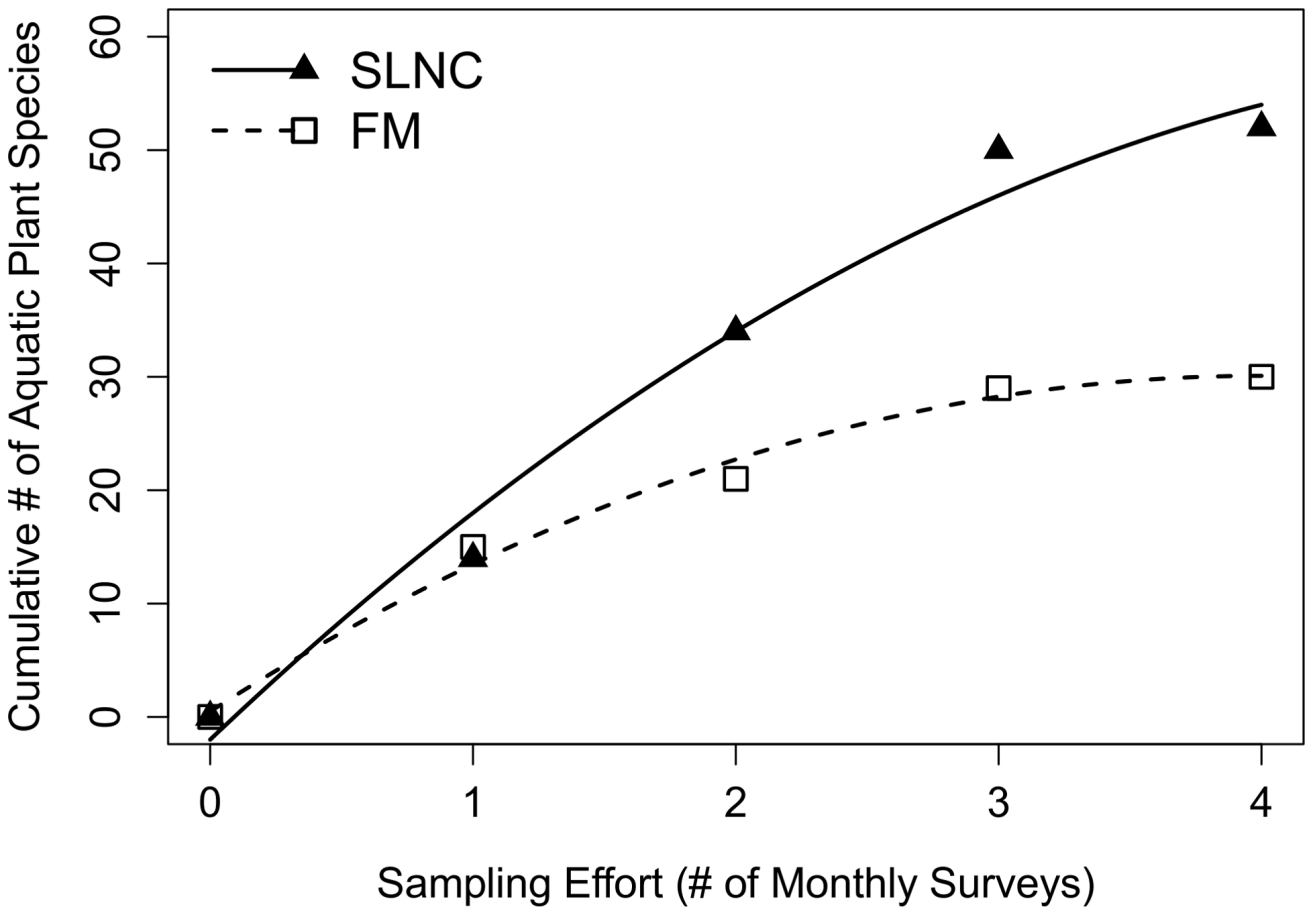
Species accumulation curves of aquatic vegetation for the two study sites in 2010. After 4 months of vegetative surveys the number of new species being found at Fort Mifflin (FM) had leveled off while at Silver Lake Nature Center (SLNC) the number of new species had not leveled off. Additional sampling at FM would likely not have found many new species while at SLNC additional sampling would likely result in higher species richness.

Aquatic habitat at SLNC was 5.75 times larger than that at FM. Plant species richness at SLNC was 1.26 times greater than at FM and plant species diversity using the Shannon-Wiener Diversity Index was 1.45 times greater at SLNC (Table 1). After four monthly surveys the cumulative number of species surveyed at FM had leveled off while at SLNC the number of species was still increasing (Figure 1). Species documented at each wetland are presented in Table S1.

### Stable Isotope Values

At SLNC there were significant differences between species for δ^13^C and δ^15^N values for all turtle tissues representing short term and long term diets (Table 2). At FM no significant differences in δ^13^C and δ^15^N values existed for turtle tissue that represented short term diets (Plasma/RBC) (Table 2). In 2008 and 2009 there were significant differences in δ^13^C values in turtle tissues that represented long term diets (tail/shell filings), with red-eared slider turtles having significantly higher δ^13^C values in 2008 and significantly lower values in 2009. In 2009 there was a significant difference in δ^15^N values from turtle tissues that represented long term nitrogen assimilation with red-eared slider turtles having significantly higher δ^15^N values (Table 2). At FM δ^13^C values of plant tissue ranged between −28.5‰ and −16.5‰ and δ^15^N values ranged between 1.03‰ and 10.09‰ (Table 3). At SLNC δ^13^C values of plant tissue ranged between −28.24‰ and −19.92‰ and δ^15^N values ranged between 0.20‰ and 11.02‰ (Table 3).

### Lipid Values

Percent lipids of tail tissue were found to be low, with a mean of 1.24%, lipid for all samples, n = 6. Red-bellied turtles had a mean of 1.32% and standard deviation of 1.06, n = 3, while red-eared slider turtles had a mean percent lipid of 1.15% and a standard deviation of 0.62, n = 3. A two-tailed t-test showed no significant difference between species (*p* = 0.8).

## Discussion

### Potential for competition in different wetlands

To our knowledge this is the first study comparing the isotopic niches of native and introduced species at different sites with measured differences in ecological characteristics. At our study sites anthropogenic impacts resulted in different habitat patch sizes. Historically, both of our study sites were either tidal creeks/floodplains (SLNC) or associated tidal wetlands of the Delaware River (FM). However, anthropogenic activities created impoundments and protected habitat at SLNC while they degraded the wetlands at FM to remnant impounded patches. These anthropogenic impacts may be the driving force behind our findings that at SLNC the δ^13^C and δ^15^N niches of red-bellied turtles and red-eared slider turtles did not overlap while the δ^13^C and δ^15^N niches did overlap at FM. In anthropogenic altered habitats, shifts in the δ^15^N niche of sailfin mollies (*Poecilia latipinna*) led to reduced growth rates in the altered habitat [29].

**Figure 2:**
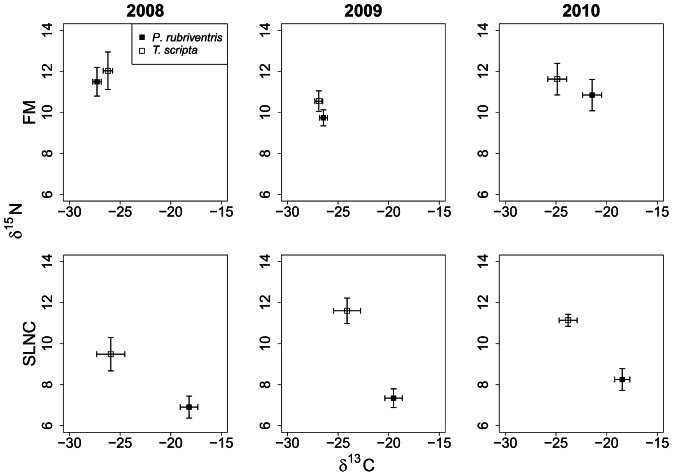
δ^13^C (x-axis) and δ^15^N (y-axis) results for blood plasma sampled from red-bellied turtles (closed squares) and red-eared slider turtles (open squares) at the Silver Lake Nature Center (SLNC, bottom row) and Fort Mifflin (FM, top row) for the years 2008, 2009 and 2010. Error bars represent the standard error of the mean. At SLNC there were significant differences for δ^13^C and δ^15^N across all three years. At FM no significant differences were found in δ^15^N values and in 2008 and 2009 no significant differences in δ^13^C values were found.

At SLNC the potential for competition for dietary resources was low as the extent of dietary resource overlap was low. The partitioned δ^13^C and δ^15^N niches were likely a factor of larger wetland size, greater vegetative species richness and greater vegetative species diversity which enabled a wider niche base for species to partition. Another potential factor impacting the isotopic niches may have been invertebrate species richness and diversity. We recognize that animal matter is important to turtle diets but red-bellied turtles and red-eared slider turtles are primarily herbivorous and are known to feed on animal matter opportunistically [40], [50]. Higher δ^15^N levels (Figure 2) of red-eared slider turtles at SLNC may have indicated that animal matter was an important driver of the dietary niche partitioning found at SLNC. Fecal sample examination from both species indicated a tenfold increase in the percent volume of animal matter in the diets of red-eared slider turtles compared to red-bellied turtles at SLNC (Pearson, unpublished data). The higher volume of animal matter in red-eared slider turtle diets is reflected by the significantly greater δ^15^N values compared to red-bellied turtles at SLNC (Table 2/Figure 2, SLNC).

At FM the potential for competition in the short term was greatly increased as the δ^13^C and δ^15^N niche axes did not significantly differ between species. Whether or not red-bellied turtles are weaker competitors than red-eared slider turtles for limited resources is yet to be determined. However, when a shared dietary resource between red-bellied turtles and red-eared slider turtles becomes limiting, competition will occur and the species better suited to obtain that resource will negatively impact the growth, fecundity or survivorship of the weaker competitor [7]. Our study showed that under certain conditions (i.e. in smaller wetlands) the potential for competition between red-bellied turtles and red-eared slider turtles did exist. If overlap for resources occurs over extended periods of time it is likely that these species will compete for resources and that this competition will have negative impacts on long term population growth of one of the species [6].

### Differences in Wetland Characteristics

At SLNC stable isotope signatures indicated that red-eared slider turtles and red-bellied turtles did not utilize the same dietary resources on either a short term or long term basis. This was consistent between years for all tissues sampled. The high vegetative species richness enabled these species to partition diets by consuming different plants at SLNC. At FM stable isotope signatures revealed no significant differences between diets of the two turtle species on a short term basis but indicated differences on a long term basis. These differences between wetland complexes can be due to several factors. One explanation could be that the range of available carbon and nitrogen stable isotopes at FM was narrower. However, this was not the case as the breadth of stable isotope values at FM was not collapsed in comparison to SLNC (Table 3). For the same set of plant species the widest breadth of carbon and nitrogen stable isotope values was found for vegetation sampled from FM. A second explanation could be differences in wetland size. Aquatic habitat available at SLNC was 5.75 times the size of aquatic habitat at FM (Table 1). Smaller habitat size reduces space available to forage which can increase the likelihood that two species will consume the same resources. At FM the depressed niche differentiation between species may have been due in part to a reduction in available habitat. A third possibility for differences in long term dietary niche overlap between wetland complexes could have been differences in dietary resources available. Adult red-bellied turtles and red-eared slider turtles are primarily herbivorous but will eat available animal material [40]. At FM the overlap for diets was due in part to FM having fewer plant species to partition (Table 1) while at SLNC the red-eared slider turtles added invertebrates to their diet causing a greater separation in dietary niches between the species.

Our research occurred at two wetland complexes that represented different disturbance histories. We recognize that we did not replicate these studies in other wetlands with similar sizes and ecological characteristics. However, our results are valid as an example of how wetland characteristics can impact the assimilation of an introduced species into native communities with different disturbance histories. This “natural experiment” [3], [51] was designed to determine how wetland characteristics relate to dietary niche overlap between red-eared slider turtles and red-bellied turtles. An increase in vegetative species richness, like that seen at SLNC, may enable red-bellied turtles and red-eared slider turtles to partition dietary resources while a narrower resource base, like that seen at FM, may lead to an increase in dietary resource overlap. Our findings are similar to those of Luiselli *et al.* who report that differences in diets of the west African mud turtle (*Pelusios castaneus*) and the west African black turtle (*Pelusios niger*) at a pristine site and an oil-polluted site in the Nigeria Delta are due to a change in dietary resource availability at the disturbed site [52]. Similarly, Kamler *et al.* report that diets of swift foxes (*Vulpes velox*) are altered based on resource availability in continuous and anthropogenically altered prairie habitats [53].

### Long-term Carbon and Nitrogen Isotopic Niche Partitioning at FM

Over the three year period of our study, red-bellied turtles and red-eared slider turtles at FM consistently overlapped in short term diets but their long term diets differed in the δ^13^C and δ^15^N values. These data suggest that the turtle populations may be highly transient. This is consistent with findings of inter-wetland movement by marked animals from FM [54]. Since short term δ^13^C and δ^15^N values overlapped but long term did not, these species were feeding on similar resources while at FM but had different diets while in other wetlands. Due to the small size of these wetlands it is likely that turtles did not reside in these wetlands for their full lifetime. Therefore, the δ^13^C and δ^15^N represent long term net diet assimilated from other habitats. Our study site at FM was adjacent to the Delaware River which may have provided access to a broader watershed for immigrating turtles to find the site or for emigrating turtles to disperse. In addition to the Delaware River acting as a source or sink of turtles for our study site there was a mosaic of remnant wetlands dotting the landscape between our study site and the John Heinz National Wildlife Refuge [54]. These wetlands may also have acted as a source or sink for turtles to/from our study site.

Alternate explanations are that red-eared slider turtle long term diet assimilation may be representative of a history of living in captivity or different responses to high protein ephemeral resources. If the red-eared slider turtles that we sampled were released pets we would expect their long term stable isotope signatures to reflect the higher protein signature of domestic turtle food or human food rather than that of wild turtle populations. If red-eared slider turtles respond more rapidly to ephemeral protein sources such as carrion or fluxes of insect larvae their long term isotopic signatures would also reflect a higher protein diet. As seen in Table 2 the nitrogen signature for tail tissue of red-eared slider turtles was higher than those for red-bellied turtles indicating greater rates of protein consumption by these turtles.

### Conservation Implications

The potential for competition between species can increase as anthropogenic impacts become more severe [4]–[6]. When competition occurs between species the negative impacts are not immediate [55] and in long lived species, such as turtles, would likely result in reduced growth rates and decreased body condition [33]. Shifts in growth rates and body condition of turtles can lead to delayed maturity and decreased lifetime fecundity [56]–[58], in turn negatively affecting population size and growth [59], [60]. If red-eared slider turtles negatively impact red-bellied turtles in Pennsylvania or native species elsewhere, then their introduction may have long term consequences on the structure of turtle communities worldwide. The continued introduction of red-eared slider turtles may lead to decreased population size or extirpation of native turtle species. As a cautionary measure the sale and release of red-eared slider turtles should be prohibited outside their native range while pre-existing owners should be required to register existing pets to further reduce the number of released animals. If continued introductions of red-eared slider turtles are prevented, then targeted control programs may be successful at stemming this species continued invasion.

## Supporting Information

Table S1 Plant species documented during the 2010 resource availability surveys. Plant species found only at FM and SLNC are on the left and right, respectively, while species found at both wetlands are in the center. We documented 31 species at FM and 51 species at SLNC.
(DOCX)
