# Supplementary material for: Stable Isotopes of C and N Reveal Habitat Dependent Dietary Overlap between Native and Introduced Turtles Pseudemys rubriventris and Trachemys scripta
Source: PLoS One. 2013 May 13;8(5):e62891. doi: 10.1371/journal.pone.0062891 (PMC3652855; doi:10.1371/journal.pone.0062891)
Supplement: Table S1 — Plant species documented during the 2010 resource availability surveys. Plant species found only at FM and SLNC are on the left and right, respectively, while species found at both wetlands are in the center. We documented 31 species at FM and 51 species at SLNC. (DOCX) [file pone.0062891.s001.docx]

| **FM** | | **FM/SLNC** | | **SLNC** | |
| --- | --- | --- | --- | --- | --- |
| **Species** | **Common Name** | **Species** | **Common Name** | **Species** | **Common Name** |
| *Amelanchier stolonifera* | Running serviceberry | *Amorpha fruticosa* | False indigo | *Acer negundo* | Black Maple |
| *Cicuta maculata* | Spotted water hemlock | *Boehmeria cylindrica* | Smallspike false nettle | *Acer rubrum* | Red maple |
| *Cornus spp.* | Dogwood | *Carex spp.* | Sedge | *Acer saccharlnum* | Sugar maple |
| *Cyperuse sculentus* | Yellow nutsedge | *Cephalanthus occidentalis* | Buttonbush | *Alnus serrulata* | Hazel alder |
| *Habiscus moscheutos* | Swamp rosemallow | *Helianthus spp.* | Sunflower | *Asclepias spp.* | Milkweed |
| *Lilium spp.* | Lily | *Lemna minor* | Duckweed | *Betula nigra* | River birch |
| *Olmus procera* | English elm | *Lythrum salicaria* | Purple loosestrife | *Bidens spp.* | Beggar-ticks |
| *Rubus spp.* | Bramble | *Myriophyllum spp.* | Water milfoil | *Callitriche palustris* | Water-starwort |
| *Solanum dulcamara* | Bittersweet nightshade | *Nuphar advena* | Spatterdock | *Celastrsi orbiculatus* | Oriental bittersweet |
| *Typha spp.* | Cattail | *Nymphaea odorata* | Fragrant water-lily | *Cornus amomum* | Silky dogwood |
| Table S1. Plant species documented during the 2010 resource availability surveys. Plant species found only at FM and SLNC are on the left and right, respectively, while species found at both wetlands are in the center. We documented 31 species at FM and 51 species at SLNC. |  | *Onoclea sensibilis* | Sesitive fern | *Cornus florida* | Flowering dogwood |
|  |  | *Parthenocissus quinquefolia* | Virginia Creeper | *Cuscata spp.* | Dodder |
|  |  | *Peltandra virginica* | Arrow arum | *Elymus spp.* | Wild rye |
|  |  | *Perscicaria sagittata* | Arrowleaf tearthumb | *Euonymus spp.* | Spindle tree |
|  |  | *Phragmites australis* | Common reed | *Gleditsia triancanthos* | Honey locust |
|  |  | *Polygonum hydropiper* | Marshpepper knotweed | *Lonicera japonica* | Japanese honeysuckle |
|  |  | *Rhamnus spp.* | Buckthorn | *Lyngbia spp.* | Filamentous algae |
|  |  | *Salix nigra* | Black willow | *Magnolia tripetala* | Umbrella tree |
|  |  | *Viburnum dentatum* | Arrowwood | *Morus rubra* | Red mullberry |
|  |  | *Vitas vulpina* | Frostgrape | *Pinus strobus* | Eastern white pine |
|  |  | *Wolffia spp.* | Watermeal | *Platanus occidentalis* | American sycamore |
|  |  |  |  | *Potamegeton confervoides* | Tuckerman's pondweed |
|  |  |  |  | *Quercus palustris* | Pin oak |
|  |  |  |  | *Rhus typhina* | Staghorn sumac |
|  |  |  |  | *Rosa multiflora* | Multiflora rose |
|  |  |  |  | *Spiraea spp.* | Steeplebush |
|  |  |  |  | *Syringa spp.* | Lilac |
|  |  |  |  | *Tilia americana* | American basswood |
|  |  |  |  | *Ulmus americana* | American elm |
|  |  |  |  | *Valeriana spp.* | Valerian |
